# Anterior lumbar interbody fusion versus transforaminal lumbar interbody fusion for correction of lumbosacral fractional curves in adult (thoraco)lumbar scoliosis: A systematic review

**DOI:** 10.1016/j.xnsj.2023.100299

**Published:** 2023-11-30

**Authors:** Daniel D. Cummins, Aaron J. Clark, Munish C. Gupta, Alekos A. Theologis

**Affiliations:** a Department of Orthopaedic Surgery, University of California — San Francisco (UCSF), 500 Parnassus Ave, MUW 3rd Floor, San Francisco, CA, 94143 United States; b Department of Neurological Surgery, UCSF, 400 Parnassus Ave, Eighth Floor, San Francisco, CA 94143, United States; c Department of Orthopaedic Surgery, Washington University, Campus Box 8233, 660 Euclid Avenue, Saint Louis, MO 63110, United States

**Keywords:** Lumbosacral, Fractional curve, Spinal deformity, Scoliosis, ALIF, TLIF, Lumbar lordosis, Coronal alignment

## Abstract

**Background:**

Anterior lumbar interbody fusion (ALIF) or transforaminal lumbar interbody fusion (TLIF) may be used to correct the lumbosacral fractional curve (LsFC) in de novo adult (thoraco) lumbar scoliosis. Yet, the relative benefits of ALIF and TLIF for LsFC correction remain largely undetermined.

**Purpose:**

To compare the currently available data comparing radiographic correction of the LsFC provided by ALIF and TLIF of LsFC in adult (thoraco)lumbar scoliosis.

**Methods:**

A systematic review was performed on original articles discussing fractional curve correction of lumbosacral spinal deformity (using search criteria: “lumbar” and “fractional curve”). Articles which discussed TLIF or ALIF for LsFC correction were presented and radiographic results for TLIF and ALIF were compared.

**Results:**

Thirty-one articles were returned in the original search criteria, with 7 articles included in the systematic review criteria. All 7 articles presented radiographic results using TLIF for LsFC correction. Three of these articles also discussed results for patients whose LsFC were treated with ALIFs; 2 articles directly compared TLIF and ALIF for LsFC correction. Level III and level IV evidence indicated ALIF as advantageous for reducing the coronal Cobb angle of the LsFC. There were mixed results on relative efficacy of ALIF and TLIF in the LsFC for restoration of adequate global coronal alignment.

**Conclusions:**

Limited level III and IV evidence suggests ALIF as advantageous for reducing the coronal Cobb angle of the LsFC in de novo adult (thoraco) lumbar scoliosis. Relative efficacy of ALIF and TLIF in the LsFC for restoration of global coronal alignment may be dictated by several factors, including directionality and magnitude of preoperative coronal deformity. Given the limited and low-quality evidence, additional research is warranted to determine the ideal interbody support strategies to address the LsFC in adult (thoraco) lumbar scoliosis.

## Introduction

In de novo adult (thoraco)lumbar scoliosis, a major lumbar curve (MC) and lumbosacral fractional curve (LsFC) characterize the deformity. While the LsFC is often thought to be a compensatory mechanism to the MC, the LsFC may also be the primary driver of the MC and global spinal malalignment in a subset of patients [1]. The LsFC can produce significant morbidity secondary to radiculopathy, which occurs from the compression of the L4, L5, and/or S1 nerve roots in a documented 80% of patients [2]. In select cases, LsFC-associated radiculopathy may be the most symptomatic aspect of deformity and addressed independently of the MC [3].

In addition to improvement of radicular leg pain, surgical correction of the LsFC has been shown to improve postoperative coronal and sagittal malalignment [4,5]. An uncorrected or under corrected LsFC may play a significant role in residual regional and global coronal and sagittal spinal imbalance and lead to surgically challenging revision procedures after MC correction [6]. As such, there is a need to determine the optimal surgical strategy to correct the LsFC coronally and sagittally in patients undergoing surgical correction of de novo adult (thoraco) lumbar scoliosis.

Interbody fusion to correct the LsFC may be accomplished using either transforaminal lumbar interbody fusion (TLIF) or anterior lumbar interbody fusion (ALIF). Both techniques can provide radiographic and symptomatic success [4,5], with evidence that interbody support can improve LsFC correction compared to no interbody support [5]. Given the importance of successful LsFC correction to symptomatic outcome, spinal realignment, and risk of reoperation, this systematic review aims to characterize and compare the relative radiographic correction of the LsFC between TLIF and ALIF across the literature.

## Methods

A systematic review was conducted following the Preferred Reporting Items for Systematic Reviews and Meta-Analyses (PRISMA) 2020 guidelines [7] (Figure, Supplementary Table 1). PubMed, SCOPUS, and MEDLINE databases were queried for the terms "lumbar" and "fractional curve" to maximize yielded articles for initial review. Last date of the search was September 24, 2022. Inclusion criteria were studies with 5 or more patients treated with TLIF or ALIF for LsFC in adults (age ≥ 18 years old) with de novo (thoraco)lumbar spinal deformity.

**Figure:**
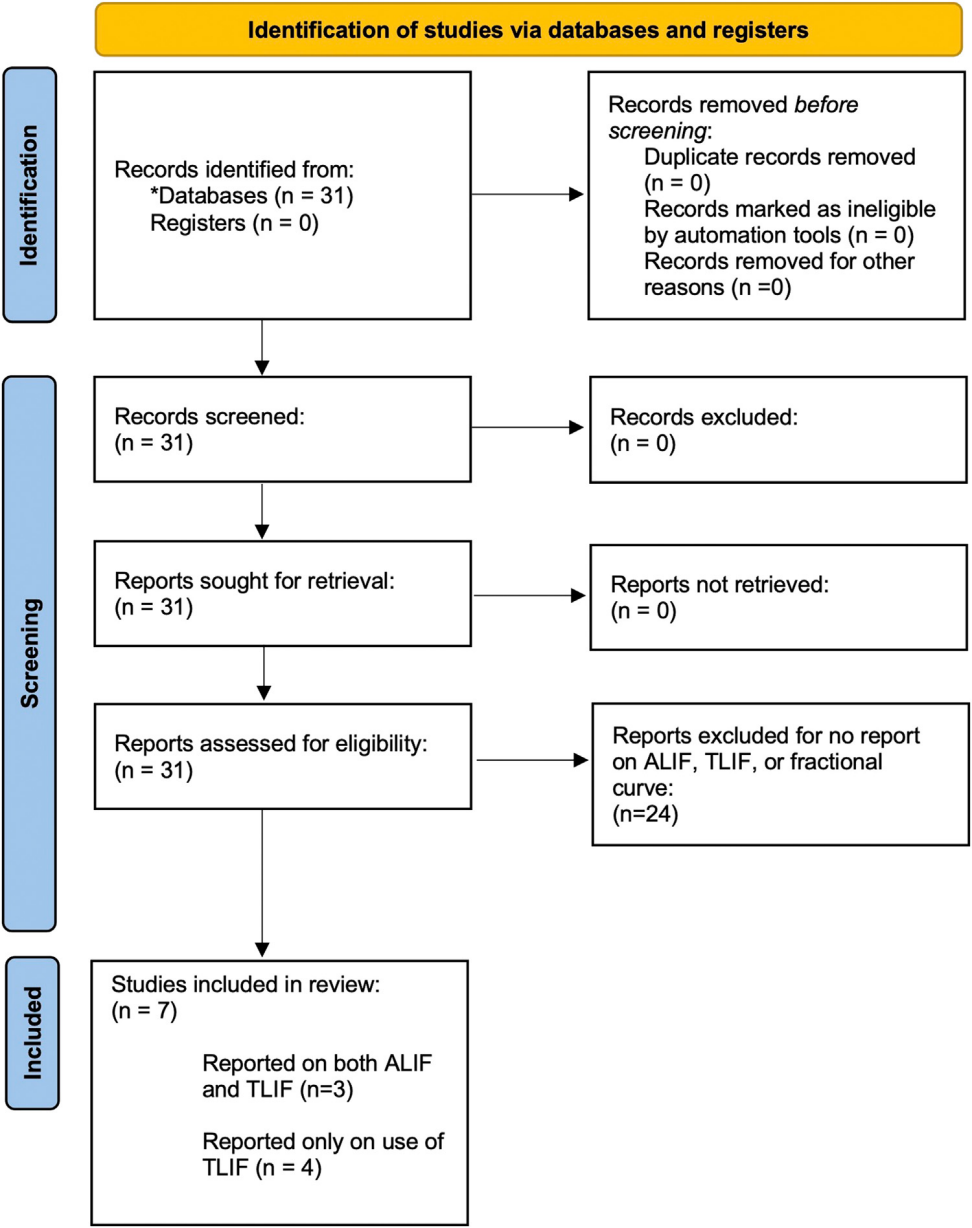
PRISMA flowchart of systematic review search criteria and results. *All articles identified in PubMed. ALIF, anterior lumbar interbody fusion; TLIF, transforaminal lumbar interbody fusion.

Only articles including radiographic parameters in the coronal or sagittal planes were included. Radiographic parameters of the coronal plane assessed included global coronal alignment (defined as distance from the central sacral vertical line [CSVL] to the C7 plumb line in the anterior-posterior plane), Cobb angle of the LsFC, and Cobb angle of the lumbar scoliosis/MC. Radiographic parameters of the sagittal plane assessed included C7 sagittal vertical axis (SVA), pelvic tilt (PT), pelvic incidence (PI), lumbar lordosis (LL), and PI-LL mismatch. Included articles must have been published between 1998 and 2022.

From these initial results, all articles were manually read to determine articles that focused on the correction of coronal and sagittal parameters of LsFC in de novo adult lumbar scoliosis. Exclusion criteria from these articles included those with pediatric patients (<18 years old) and those not including at least 1 coronal and sagittal lumbosacral parameter both before and after surgery. Articles were excluded if only a subset of patients received TLIF or ALIF at the LsFC and radiographic data were not presented for this subset of patients. Only original research was included; systematic reviews and meta-analyses on the topic were discussed in the manuscript but excluded from the systematic review.

Two independent reviewers independently assessed all included studies. Level of evidence was determined based on previously defined categories used in the surgical literature [8]:

Level I: High-quality prospective cohort study with adequate power or systematic review of these studies.
Level II: Lesser-quality prospective cohort, retrospective cohort study, untreated controls from an Randomized Controlled Trial, or systematic review of these studies.
Level III: Case-control study or systematic review of these studies.
Level IV: Case series.
Level V: Expert opinion; case report or clinical example; or evidence based on physiology, bench research, or “first principles.”

## Results

Initial database queries using the described criteria yielded 31 resulting manuscripts (Figure). A total of 7 studies were included describing radiographic results using TLIF and/or ALIF for LsFC correction of de novo adult (thoraco)lumbar scoliosis. A total of 252 patients were treated with TLIF and 134 with ALIF for LsFC correction. All studies were retrospective cohort studies, giving only level III and level IV evidence [8]. All 7 studies reported results for patients who received TLIF; 4 of these studies reported using only TLIF and 3 also reported on patients who received ALIF for LsFC correction. Two of these studies directly compared TLIF with ALIF and 1 reported results for each without direct comparison (Table 1).

**Table 1:** All studies reporting radiographic outcomes using TLIF or ALIF for lumbosacral fractional curve correction.

Study #	Author	Year	TLIF (# patients)	ALIF (# patients)	TLIF versus ALIF directly compared
1	Buell et al. [9]	2021	47	59	yes
2	Amara et al. [10]	2020	23	44	yes
3	Geddes et al. [11]	2021	9	31	no
4	Theologis et al. [5]	2021	58	0	no
5	Bao et al. [13]	2019	14	0	no
6	Ha et al. [14]	2021	13	0	no
7	Zhang et al. [17]	2020	77	0	no
8	Manwaring et al. [15]	2014	11	0	no
All		N/A	252	134	N/A

ALIF, anterior lumbar interbody fusion; FC, fractional curve; TLIF, transforaminal lumbar interbody fusion.

There were no systematic reviews and no meta-analyses comparing ALIF and TLIF for correction of the LsFC. All included studies had risks of bias including retrospective design with lack of randomization or blinding, selective reporting, low sample sizes without performance of power calculations, and lack of direction comparisons between treatment groups (with the exception of 2 studies).

### Studies reporting use of TLIF and ALIF for lumbosacral fractional curve correction

There were 3 level III studies that reported on TLIF and ALIF for LsFC correction. Buell et al*.* and Amara et al. performed direct comparisons between TLIF and ALIF for correction of LsFC [9,10]. Buell et al*.* [9] compared TLIF and ALIF with both an unmatched analysis and propensity-matched analysis for a number of interbody fusions above L4, TLIF/ALIF at L4–L5, TLIF/ALIF at L5–S1. In the propensity-matched analysis controlling for the aforementioned variables, there was no significant difference between TLIF and ALIF at L4–S1 in any radiographic variable of interest. These included (TLIF vs. ALIF): pelvic obliquity (2.3^0^ ± 1.9^0^ vs. 1.4^0^ ± 1.3^0^; p = .113), MC coronal Cobb angle (20.5^0^ ± 14.6^0^ vs. 24.2^0^ ± 15.1^0^; p = .330), LsFC coronal Cobb angle (6.8^0^ ± 5.4^0^ vs. 7.4^0^ ± 5.4^0^; p = .755), C7–

S1 SVA (2.8  ± 5.7cm vs. 3.0  ± 5.7cm; p = .917), PT (23.8^0^ ± 9.3^0^ vs. 23.9^0^ ± 8.2^0^; p = .962), PI-LL mismatch (−0.1^0^ ± 13.3^0^ vs. 4.6^0^ ± 11.6^0^; p = .164), and TK (−53.4^0^ ± 14.5^0^ vs. −50.9^0^ ± 19.1^0^; p = .584) (Table 2).

**Table 2:** Studies reporting radiographic outcomes for use of both TLIF and ALIF for lumbosacral fractional curve correction.

Study	Buell et al. [9]	Amara et al. [10]	Geddes et al. [11]
	TLIF versus ALIF, for matched analysis	TLIF versus ALIF	TLIF versus ALIF
Level of evidence	III	III	III
Total patients (n)	28 versus 28	23 versus44	9 versus31
Interbody levels fused L3–L4 (%) L4–L5 (%) L5–S1 (%)	0 17 (60.7%) versus 14 (50.0%); p = .420 14 (50.0%) versus 10 (35.7%), p = .280	N/A N/A N/A	N/A N/A N/A
Follow-up (months)	24.0	35.8 (12–150)	N/A
Cobb FC (°) Pre-op Post-op Change	20.4 ± 7.6 versus 21.0 ± 6.6; p = .611 6.8 ± 5.4 versus 7.4 ± 5.4; p = .755 NA; p = < .001 versus p < .001	16.0 versus 17.2; p = .33 7.0 versus 5.6; p = .18 9.0 versus 11.7; p = .067	10.3 ± 6.8 versus 18.3 ± 9.3; p = N/A 7.1 ± 4.2. versus 6.1 ± 5.3; p = N/A 3.2 ± 3.4 versus 12.1 ± 6.0; p = N/A
Major Cobb (°) Pre-op Post-op Change	52.8 ± 19.0 versus 53.9 ± 12.5; p = .394 20.5 ± 14.6 versus 24.2 ± 15.1; p = .330 NA; p < .001 versus p < .001	32.4 versus 34.3; p = .62 20.0 versus 16.5; p = .21 12.4 versus 17.9; p = .17	ALIF only: 40.6 ± 20.8 17.1 ± 12.4 23.5 ± 14.7
CSVL (cm) Pre-op Post-op Change	3.0 ± 2.0 versus 3.6 ± 2.9; p = .806 3.0 versus 2.6 ± 2.1; p = .646 NA; p = 0.820 versus p = .111	2.2 versus 2.9; p = 33 1.5 versus 2.6; p = .029 -0.72 versus -0.38; p = .61	ALIF only: 3.3 ± 2.16 2.3 ± 1.6 1.0 ± 2.4
C7–S1 SVA (cm) Pre-op Post-op Change	4.9 ± 6.6 versus 6.9 ± 6.4; p = .267 2.8 ± 5.7 versus 3.0 ± 5.7; p = .917 NA; p = 0.071 versus p = .011	4.8 versus 6.0; p = .37 4.8 versus 4.7; p = .92 0.015 versus -1.3; p = .38	ALIF only: 9.1 ± 5.1 5.0 ± 3.4 4.0 ± 4.8
PT (°) Pre-op Post-op Change	25.8 ± 7.9 versus 24.7 ± 7.7; p = .601 23.8 ± 9.3 versus 23.9 ± 8.2; p = .962 NA; p = .222 versus p = .598	26.6 versus 25.5; p = .70 27.3 versus 24.5; p = .24 1.9 versus -2.1; p = .127	ALIF only: 28.2 ± 9.5 N/A N/A
PI-LL mismatch (°) Pre-op Post-op Change	16.4 ± 17.2 versus 18.5 ± 15.2; p = .641 −0.1 ± 13.3 versus 4.6 ± 11.6; p = .164 N/A; p < .001 versus p < .001	18.3 versus 23.1; p = .23 19.2 versus 14.0; p = .20 N/A	N/A N/A N/A
LL Pre-op Post-op Change	29.2 ± 17.2 versus 34.6 ± 19.2; p = .276 50.8 ± 11.8 versus 52.4 ± 13.0; p = .635 N/A, p < .001 versus N/A, p < .001	43.6 versus 32.9; p = .016 42.7 versus 42.4; p = .93 -0.9 versus 9.1; p = .028	ALIF Only: 31.0 ± 14.4 48.2 ± 15.2 17.2 ± 15.5
TK (°) Pre-op Post-op Change	−31.7 ± 17.8 versus −32.7 ± 19.5; p = .85 −53.4 ± 14.5 versus −50.9 ± 19.1; p = .58 N/A	N/A N/A N/A	N/A N/A N/A

ALIF, anterior lumbar interbody fusion; TLIF, transforaminal lumbar interbody fusion; FC, fractional curve; CSVL, central sacral vertical line; SVA, sagittal vertical axis; PT, pelvic tilt; PI, pelvic incidence; LL, lumbar lordosis.

While Buell et al. [9] found no significant differences in postoperative global coronal alignment between patients who were treated with TLIF in the LsFC (3.0 ± 2.5 cm) and patients whose LsFC were addressed with ALIF (2.6 ± 2.1 cm) (p = .646), it was found that there was significant reductions in coronal malalignment with ALIFs (from 3.6 ± 2.9 cm preoperatively to 3.0 ± 2.1 cm postoperatively), which was not seen with TLIFs (from 3.0 ± 2.0 cm preoperatively to 3.0 ± 2.5 cm postoperatively), thus favoring ALIF. In contrast, Amara et al*.* [10] compared patients who received TLIFs in the LsFC and patients whose LsFC were corrected with ALIF and found a significant difference in magnitude of residual postoperative global coronal imbalance [CSVL 1.5 cm (TLIF) vs. 2.6 cm (ALIF); p = .029] and increase from preoperative to postoperative LL [-0.87^0^ (TLIF) vs. 9.1^0^ (ALIF); p = .028] (Table 2).

While Geddes et al*.* [11] did not directly compare TLIF versus ALIF for LsFC correction, they reported results for the use of TLIF and ALIF separately. Compared to TLIF, ALIF had a greater degree of improvement of LsFC coronal Cobb angle (TLIF: 3.2^0^ ± 3.4^0^ vs. ALIF: 12.1^0^ ± 6.0^0^; p = N/A). No statistical comparison was made between TLIF and ALIF in this study. However, Geddes et al*.* [11] did compare change in LsFC Cobb angles for patients who received TLIF (3.2^0^ ± 3.4^0^) to posterior instrumented fusion without interbody support of the LsFC (no TLIF, 5.5^0^ ± 4.8^0^) and found no significant difference (p = .144), with a trend toward greater LsFC reduction without TLIF. In contrast, a comparison of patients treated with ALIF compared to posterior instrumented fusion without interbody support of the LsFC found significantly greater reduction in LsFC coronal Cobb angles in patients treated with ALIF compared to posterior spinal fixation alone (ALIF: 12.1^0^ ± 6.0^0^ vs. without ALIF: 4.8^0^ ± 4.5^0^; p = .000). These 2 indirect comparisons would therefore favor ALIFs in attaining a greater degree of coronal correction of the LsFC compared to TLIFs [11].

### Studies reporting only use of TLIF for fractional curve correction

Four level IV studies reported only on the use of TLIF for LsFC correction (Table 3). Theologis et al*.* [5] reported on de novo adult (thoraco)lumbar scoliosis patients treated with posterior-only operations with or without TLIFs of the LsFC. The average LsFC correction was noted to be 17.3° using TLIF but did not specify additional parameters for the subset of patients who received TLIF specifically. Theologis et al*.* [5] also described outcomes by coronal malalignment types (A vs. B vs. C as defined by Bao et al. [12]. In this 3-part classification system, balance is defined by magnitude relative to the CSVL and directionality of the alignment relative to the convexity of the major lumbar scoliosis: type A = SVL < 3 cm; type B = CSVL > 3 cm contralateral to the convexity of the major lumbar scoliosis; and =type C = CSVL > 3 cm ipsilateral to the convexity of the major lumbar scoliosis [12]. It was noted that the type C patients had significantly greater magnitudes of the LsFC [5]. Of the type C patients preoperatively, it was found that 67% of them remained type C (unbalanced) postoperatively [5]. These patients, compared to type C patients who converted to type A after surgery, had significantly greater preoperative LsFC, greater pre-op coronal Cobb angles, and more commonly involved TLIFs of the LsFC [5]. Additionally, use of TLIFs provided better correction of LsFC compared to no interbody support [5].

**Table 3:** Studies reporting radiographic outcomes for use of only TLIFs for lumbosacral fractional curve correction.

Study	Theologis et al., [5]	Bao et al., [13]	Ha et al. [14]	Manwaring et al. [15]
Level of evidence	IV	IV	IV	IV
Total patients (n)	58	14	13	11
Interbody levels fused L3–L4 (%) L4–L5 (%) L5–S1 (%)	18 (31.0) 47 (81.0) 51 (87.9)	N/A N/A 14 (100)	1 (7.7) 6 (46.2) 11 (84.6)	N/A N/A N/A
Follow-up (months)	43.2 ± 17.2	12.0	N/A	22.9 (6 - 37.1)
Cobb of FC (°) Pre-op Post-op Change	N/A N/A 17.8 ± N/A	N/A N/A N/A	18.4 ± 11.8 7.6 ± 7.8 N/A; p = .0089	9.2 4.1 N/A; p < .009
Major Cobb (°) Pre-op Post-op Change	N/A N/A N/A	50.5 ± 20.6 29.5 ± 16.5 N/A	57.4 ± 21.7 23.5 ± 11.5 N/A; p < .0001	28.8 12.8 N/A; p < .0001
CSVL (cm) Pre-op Post-op Change	N/A N/A N/A	2.9 ± 1.9 1.2 ± 5.7 N/A	N/A N/A N/A	1.8 1.6 N/A; p > .05
C7–S1 SVA (cm) Pre-op Post-op Change	N/A N/A N/A	2.7 ± 8.0 1.4 ± 1.6 N/A	5.7 ± 5.4 2.6 ± 2.4 N/A; p = .0018	3.1 4.5 N/A; p > .05
PT (°) Pre-op Post-op Change	N/A N/A N/A	24.7 ± 9.9 22.5 ± 11.3 N/A	N/A N/A N/A	22.4 26.2 N/A; p >.05
LL Pre-op Post-op Change	N/A N/A N/A	6.4 ± 29.9 30.3 ± 13.4 N/A	35.9 ± 25.3 44.5 ± 12.2 N/A; 0.18	39.5 36.7 N/A; p > .05
PI-LL mismatch (°) Pre-op Post-op Change	N/A N/A N/A	37.9 ± 23.5 12.9 ± 11.2 N/A	N/A N/A N/A	N/A N/A N/A
TK (°) Pre-op Post-op Change	N/A N/A N/A	6.8 ± 24.9 17.6 ± 9.8 N/A	N/A N/A N/A	N/A N/A N/A

ALIF, anterior lumbar interbody fusion; TLIF, transforaminal lumbar interbody fusion; FC, fractional curve; CSVL, central sacral vertical line; SVA, sagittal vertical axis; PT, pelvic tilt; PI, pelvic incidence; LL, lumbar lordosis.

Bao et al. [13] did not report LsFC parameters, but did present a number of additional sagittal alignment parameters in patients treated with TLIF for LsFC correction (Table 3**)**. Although a statistical comparison was not performed, these included significant improvements (pre-op vs. last follow-up) after surgery for the MC Cobb angles (pre-op: 50.5^0^ ± 20.6^0^ vs. post-op: 29.5^0^ ± 16.5^0^), C7-S1 SVA (pre-op: 2.7 ± 8.0cm  vs. post-op 1.4 ± 1.6 cm), and PI-LL mismatch (pre-op 37.9^0^ ± 23.5^0^ vs. post-op: 12.9^0^ ± 11.2^0^). Ha et al*.* [14] demonstrated significant improvements (pre-op vs. last follow-up) in LsFC Cobb angles using TLIFs (pre-op: 18.4^0^ ± 11.8^0^ vs. post-op: 7.6^0^ ± 7.8^0^; p = .0089). This study also demonstrated significant improvement in the MC Cobb angles (pre-op: 57.4^0^ ± 21.7^0^ vs. post-op 23.5^0^ ± 11.5^0^; p < .0001), global coronal alignment (CSVL) (pre-op 29.4 mm ± 18.8 mm vs. post-op 12.1 mm ± 5.7 mm) and C7-S1 SVA (pre-op: 5.7  ± 5.4 cm vs. post-op: 2.6  ± 2.4 cm; p < .0001) [14]. Manwaring et al*.* [15] also demonstrated the use of TLIF in the LsFC resulted in significant improvements in LsFC coronal Cobb angles (preop: 9.2^0^ vs. postop: 4.1^0^; p < .009).

## Discussion

The LsFCis an important driver of disability and deformity in adults with de novo (thoraco)lumbar scoliosis [1], [2], [3]. Its appropriate surgical correction is paramount for relief of lumbar radiculopathy and for adequate restoration of regional and global coronal alignment, particularly in patients with coronal imbalance ipsilateral to the convexity of the main (thoraco)lumbar scoliosis (ie, Bao type C / Obeid type 2 deformities) [12,^16^,17]. As such, there is a need to determine the optimal surgical strategy to correct the LsFC coronally in patients undergoing surgical correction of de novo adult (thoraco)lumbar scoliosis. In this systematic review, we characterized and compared for the first time the coronal radiographic correction of the LsFC between TLIF and ALIF. From 7 articles, there were 2 main findings of this study: (1) there is limited level III and IV evidence suggesting that ALIF provides better radiographic correction of the LsFC's coronal magnitude compared to TLIF and (2) there were mixed results on the comparative efficacy of restoration of postoperative global coronal alignment when ALIF or TLIF were used to address the LsFC.

For regional correction of the coronal Cobb angle of the LsFC in de novo (thoraco)lumbar scoliosis in adults, we found limited level III and IV evidence that ALIF is advantageous to TLIF. While Buell et al*.* [9] found no significant differences in LsFC coronal Cobb angle change between TLIF and ALIF, Amara et al. [10] found a trend close to significant favoring ALIF. In the study by Geddes et al. [11] it was noted that ALIF had a clinically significantly greater degree of LsFC coronal curve reduction. It is also notable that Geddes et al*.* [11] reported a significant benefit in reducing the LsFC coronal Cobb angle when adding ALIF to posterior spinal fusion alone but not with TLIF. While there was an increase noted in LsFC coronal Cobb angles using TLIF in the study by Geddes et al. [11], other studies have demonstrated that TLIFs provide better coronal correction of the LsFC when compared to no TLIF of the LsFC [5].

For restoration of global coronal alignment in patients with coronal imbalance ipsilateral to the convexity of the main (thoraco)lumbar scoliosis, the Obeid-Coronal Malalignment classification recommends surgical strategies be aimed at adequately correcting the LsFC [16,17]. Interbody and posterior releases of the LsFC are recommended for LsFCs without previously fused interbody spaces [16,17]. In our literature review, there were mixed results on comparative efficacy of restorating global coronal realignment when TLIFs or ALIFs were used to correct the LsFC. In the study by Amara et al. [10] there were significant differences in postoperative global coronal alignment between patients who received TLIFs in the LsFC (CSVL 1.5 cm) compared to patients whose LsFC were treated with ALIFs (CSVL 2.6 cm). However, it is important to note that both groups' CSVL deviations were <3 cm postoperatively, which likely translates to no clinically or functionally significant differences [18]. Furthermore, while Buell et al. [9] found no significant differences in postoperative global coronal alignment, it is noteworthy that there was a significant improvement in global coronal alignment in patients in whom ALIFs were used to address the LsFC, but not in patients in whom the LsFC was addressed with TLIFs.

Along the same vein, coronal deformities with preoperative global malalignment >3 cm ipsilateral to the direction of the lumbar MC convexity (Bao type C / Obeid type 2) were found by Theologis et al*.* to remain globally maligned in the same direction postoperatively 67% of the time with or without the use of TLIFs in the LsFC [5,^16^,17]. Despite TLIFs providing significantly better correction of the LsFC in these patients, the correction of the LsFC was still not sufficient to achieve appropriate global coronal balance [5]. Conversely, 2 prior studies (Bao et al. [12] and Ha et al. [14]) reported acceptable postoperative global coronal alignment following surgeries utilizing TLIFs for the LsFC. These conflicting data highlight that there are a number of additional factors relevant to restoring global coronal alignment beyond the interbody fusion approach to the LsFc [19,20]. These may include, but are not limited to, preoperative coronal balance, the relative magnitude of coronal correction of the main lumbar curve relative to the coronal correction of the LsFc, presence, and magnitude of thoracic scoliosis, curve flexibility, leg length discrepancy, level of upper instrumented vertebrae, and method of pelvic fixation (ie, inclusion of kickstand rod). As these factors vary considerably from case to case, future studies that adjust for these background factors (and many others) will ideally provide greater clarity on the relative utility of ALIF and TLIF of the LsFC in the correction of global coronal alignment in adults (thoraco)lumbar scoliosis.

The results of this study should be considered in the context of its limitations. An important limitation is the quality of available evidence on the topic, which was limited to level III and IV retrospective cohort studies. Importantly, there was no randomized trial to date that has specifically explored the relative efficacy of TLIF and ALIF for radiographic correction of the LsFC in de novo adult (thoraco)lumbar scoliosis. In addition to low-level evidence, there were very few studies that have been published on the relative radiographic correction of the LsFC with different interbody approaches (ie, only 3 studies reported results for both TLIF and ALIF and 2 studies directly compared both approaches). Additionally, a number of studies included in this review lacked granularity in operative detail, including precise interbody levels fused, which precluded more direct and accurate comparisons of TLIF to ALIF at different interbody levels. It is possible for instance, that benefits to each approach may be seen when used at specific interbody levels or deformity types, which could not be compared given the available literature. All these limitations highlight that this is a relatively under-investigated area of spine surgery that is ripe for further inquiry. This review will ideally bring greater attention to this important topic in adult spinal deformity surgery and will help facilitate higher-quality investigations that more clearly elucidate the ideal interbody strategy to address the LsFC in de novo adult (thoraco)lumbar scoliosis. Many avenues can be taken to further study this clinical question, including multicenter retrospective and/or prospective comparative cohort analyses with robust patient numbers that focus specifically on adults with de novo (thoraco)lumbar scoliosis treated with only ALIF or TLIF of the LsFC, document rigorously and accurately regional and global radiographic coronal parameters, and control for confounding factors that influence correction of the LsFC and global coronal alignment (ie interbody cage size/lordosis, number of levels of interbody fusions, LsFC, and main curve magnitudes and flexibility, preoperative coronal balance magnitudes and Bao/Obeid types, upper instrumented vertebrae, and pelvic fixation strategies).

## Conclusions

In this systematic review assessing relative radiographic corrections of LsFCswith ALIF compared to TLIFs, there was limited level III and IV evidence that suggested ALIF provided better radiographic correction of the LsFC's coronal magnitude compared to TLIF. There were mixed results on the comparative efficacy of restoration of postoperative global coronal alignment when ALIF or TLIF were used to address the LsFC, which may be a result of multiple factors, including directionality and magnitude of preoperative global coronal malalignment. Further higher-level evidence is needed to more clearly elucidate the ideal interbody strategy to address the LsFC in de novo adult (thoraco)lumbar scoliosis.

## Declaration of competing interest

The authors declare that they have no known competing financial interests or personal relationships that could have appeared to influence the work reported in this paper.

## Appendix Supplementary materials

Image, application 1
